# The Pharmacology of Visual Hallucinations in Synucleinopathies

**DOI:** 10.3389/fphar.2019.01379

**Published:** 2019-12-09

**Authors:** Mirella Russo, Claudia Carrarini, Fedele Dono, Marianna Gabriella Rispoli, Martina Di Pietro, Vincenzo Di Stefano, Laura Ferri, Laura Bonanni, Stefano Luca Sensi, Marco Onofrj

**Affiliations:** ^1 ^Department of Neuroscience, Imaging, and Clinical Sciences, University G. d'Annunzio of Chieti-Pescara, Chieti, Italy; ^2^Behavioral Neurology and Molecular Neurology Units, Center of Excellence on Aging and Translational Medicine—CeSI-MeT, University G. d'Annunzio of Chieti-Pescara, Chieti, Italy; ^3^Departments of Neurology and Pharmacology, Institute for Mind Impairments and Neurological Disorders—iMIND, University of California, Irvine, Irvine, CA, United States

**Keywords:** visual hallucination, dementia with Lewy bodies, Parkinson's disease, synucleinopathy, default mode network

## Abstract

Visual hallucinations (VH) are commonly found in the course of synucleinopathies like Parkinson's disease and dementia with Lewy bodies. The incidence of VH in these conditions is so high that the absence of VH in the course of the disease should raise questions about the diagnosis. VH may take the form of early and simple phenomena or appear with late and complex presentations that include hallucinatory production and delusions. VH are an unmet treatment need. The review analyzes the past and recent hypotheses that are related to the underlying mechanisms of VH and then discusses their pharmacological modulation. Recent models for VH have been centered on the role played by the decoupling of the default mode network (DMN) when is released from the control of the fronto-parietal and salience networks. According to the proposed model, the process results in the perception of priors that are stored in the unconscious memory and the uncontrolled emergence of intrinsic narrative produced by the DMN. This DMN activity is triggered by the altered functioning of the thalamus and involves the dysregulated activity of the brain neurotransmitters. Historically, dopamine has been indicated as a major driver for the production of VH in synucleinopathies. In that context, nigrostriatal dysfunctions have been associated with the VH onset. The efficacy of antipsychotic compounds in VH treatment has further supported the notion of major involvement of dopamine in the production of the hallucinatory phenomena. However, more recent studies and growing evidence are also pointing toward an important role played by serotonergic and cholinergic dysfunctions. In that respect, *in vivo* and *post-mortem* studies have now proved that serotonergic impairment is often an early event in synucleinopathies. The prominent cholinergic impairment in DLB is also well established. Finally, glutamatergic and gamma aminobutyric acid (GABA)ergic modulations and changes in the overall balance between excitatory and inhibitory signaling are also contributing factors. The review provides an extensive overview of the pharmacology of VH and offers an up to date analysis of treatment options.

## Introduction

The focus of the review concerns visual hallucinations (VH), a prominent feature of Parkinson's disease (PD) and dementia with Lewy bodies (DLB). The review is structured as a short overview of the clinical presentations, a detailed analysis of the mechanisms, neurotransmitters, and neural networks that are involved in the process (**Table 1**). Finally, the review dissects drugs that are related to the induction and resolution of VH (**Table 2**).

**Table 1:** Neurotransmitters acting on visual hallucinations (VH) onset and resolution.

Serotonergic system			
*Seven subfamilies of receptors (from 5HT1 to 5HT7)*			
**Receptor**	**Mechanism of action**	**Sites of action**	**Effects on VH**
5HT1A	Antagonism	Raphe nuclei of medulla oblongata, amygdala, and hippocampus	Induction
5HT2A	Agonism Antagonism	Prefrontal cortex, anterior and posterior cingulate cortex	Induction Inhibition
5HT2C	Agonism	Prefrontal cortex, anterior cingulate cortex, and ventral tegmental area	Induction
5HT3	Antagonism Agonism	Area postrema, nucleus *tractus solitarius*, amygdala, and dorsal raphe nucleus	Inhibition Induction
SERT	Inhibition	Caudate nucleus, putamen, posterior cingulate cortex	Inhibition
			
**Dopaminergic system**			
***Five subtypes of receptors (D1R to D5R)***			
**Receptor**	**Mechanism of action**	**Sites of action**	**Effects on VH**
D2-like (*D2R*, *D3R*)	Antagonism	Striatum, external globus pallidus, nucleus accumbens, amygdala, cerebral cortex, hippocampus, and pituitary gland	Inhibition Inhibition
**Cholinergic system**			
***Two families of receptors (muscarinic and nicotinic receptors)***			
**Receptor/substrate**	**Mechanism of action**	**Sites of action**	**Effects on VH**
mAchR	Antagonism	Striatum, thalamus, ventral tegmental area, hippocampus, and substantia nigra (pedunculopontine network and basal forebrain network)	Induction
AchE	Inhibition	CNS	Inhibition
**Glutamatergic system**			
***Two types of receptors: ionotropic glutamate receptors (AMPA, kainate, and NMDA) and metabotropic glutamate receptors (mGluR 1–8 receptors)***			
**Receptor**	**Mechanism of action**	**Sites of action**	**Effects on VH**
NMDAR	Antagonism	Ventral subiculum	Induction
**Opioid system**			
***Three types of receptors: KOR, MOR, DOR***			
**Receptor**	**Mechanism of action**	**Sites of action**	**Effects on VH**
KOR	Agonism	Hypothalamus, periaqueductal gray, and claustrum	Induction
MOR	Agonism	Cortex, hypothalamus, periaqueductal gray, striosomes, rostral ventromedial medulla	Induction

The table outlines the main sites of action, at molecular and anatomical level, of the neural networks involved in VH.

**Table 2:** The table encompasses common causes of drug-induced visual hallucinations (VH) (top), highlighting the mechanisms of action, where known.

Drugs inducing VH		
**System**	**Category**	**Drugs**
**Serotonergic**	Psychedelic	LSD Psilocin DMT
	Antidepressant	Fluoxetine Amitriptyline Clomipramine Imipramine
**Dopaminergic**	Dopamine precursor	Levodopa
	Dopamine agonist	Pramipexole Ropinirole Rotigotine
	Inhibition of dopamine catabolism *(Indirect mechanism)*	Rasagiline Selegiline
**Cholinergic**	Antimuscarinic	Atropine Hyoscyamine Scopolamine
**Glutamatergic**	Anesthetic	Ketamine Phencyclidine Propofol
	Neuroprotection *(Unclear role)*	Memantine Amantadine
**Opioid**	Psychedelic	Salvinorin-A
	Analgesic	Morphine Fentanyl Oxycodone Tramadol
**Miscellanea**	Voltage sensitive calcium channel antagonism *(Unclear mechanism)*	Verapamil Diltiazem
	Antimicrobial *(Unclear mechanism)*	Carbapenems Trimethoprim-sulfamethoxazole Voriconazole Posaconazole
**Therapeutic options for VH in synucleinopathies**		
**Category**	**Drugs**	
*Novel antiserotonergic*	Pimavanserin Ondansetron	
*Atypical antipsychotic*	Quetiapine Clozapine	

The lower part of the table shows instead suitable therapeutic options for VH in synucleinopathies.

### Clinical Setting

VH and rare hallucinations in other modalities are the hallmarks of synucleinopathies like PD, Parkinson's disease with dementia (PDD), and DLB. Explorative or consensus studies (Onofrj et al., 2002; Diederich et al., 2005; Goetz et al., 2011) have analyzed the time of appearance, prevalence, and correlation of VH with ongoing cognitive decline (Barnes et al., 2003; Ramirez-Ruiz et al., 2006; Ramirez-Ruiz et al., 2007; Barnes and Boubert, 2008) and indicated that VH are associated with and predict the presence of α-synuclein (i.e., Lewy bodies) deposits (Harding et al., 2002; Williams and Lees, 2005; Halliday et al., 2011). The phenomenon is so common in the disease progression that a brain bank-related study demonstrated that if no history of VH is found in the PD course, the diagnosis should be challenged (Williams and Lees, 2005). VH are also a core criterion for the diagnosis of DLB (McKeith et al., 2017). Upon the clinical course of PD, VH can appear as simple or complex phenomena. The original dichotomy between early-simple and late-complex presentations of VH (Fénelon et al., 2000; Barnes and David, 2001) has been nowadays discarded by evidence showing that the occurrence of VH invariably bears an unfavorable prognostic outcome, independently of the time of appearance (Goetz et al., 2006). It is nevertheless true that simple VH occur in the early stage of the disease. Simple VH are usually described as altered perceptions of blurred moving images occurring in/or outside of the visual field (extracampine hallucinations) (Chan and Rossor, 2002; Diederich et al., 2005). VH can also take the form of simple distortions of images often characterized by the association with humanized traits (eidol-idol) of elements that are present in the visual field (Ey, 1973). The phenomenon is commonly and more properly described as “illusions” or “pareidolia,” events that are the result of distorted perceptions (Uchiyama et al., 2012). VH occurring in the early course of DLB and cognitively-preserved PD patients are perceived as extraneous to reality by the hallucinating subject and they suddenly appear in the form of complex images, animals, or miniature figures (Onofrj et al., 2013) that generally disappear when the patient, employing a coping strategy, refocuses his/her attention (Diederich et al., 2009). With the disease progression and the appearance of cognitive decline, early VH that are initially associated with preserved insight are then replaced by complex VH (Fénelon, 2006). At later stages, DLB patients may be clinically indistinguishable from PDD patients who exhibit VH along with prominent cognitive impairment. Thus, clear quantitative and/or qualitative distinctions between DLB patients and PDD patients cannot be made as complex VH are the most common manifestation of both conditions. Complex VH are characterized by the loss of insight and the presence of a complex narrative structure (Onofrj et al., 2001; Fénelon, 2006). In complex VH, hallucinating images do interact and move across the visual field with kinetic properties, thereby generating enriched scenarios (i.e., fighting demons, marching warriors, multi-colored funerals, molesting presences) (Fénelon, 2006; Onofrj et al., 2006; Diederich et al., 2009; Onofrj et al., 2013). These complex VH are named paraphrenic hallucinations (Ey, 1973). Complex VH may exhibit different duration and onset time. In the early stages, they typically represent a transient phenomenon often prone to spontaneous remission. However, VH may also occur as a continuous hallucinatory state akin to a “crepuscular status” that lasts for days or until treatment is started. Although there is no substantial evidence supporting the notion of a progression from simple to complex VH occurring in the same PD patient, the appearance of VH almost always predicts, since early stages, within a definite and foreseeable time frame, the incoming occurrence of cognitive decline (Onofrj et al., 2007).

The present review also focuses on the biochemical basis of VH in hallucinatory phenomena triggered by drugs or neurological conditions. Drug-induced hallucinations are typically driven by the activation of the serotonergic system (as in the case of LSD or psilocybin) and involve sensorial modalities in addition to the visual one (Geyer and Vollenweider, 2008). Time and space dysperceptions, ego-dissolution, depersonalization, derealization, and cenesthetic hallucinations are quite common upon the consumption of psychedelic compounds (Geyer and Vollenweider, 2008; Carhart-Harris et al., 2010). However, drug-induced hallucinations may exhibit different presentations when occurring in the context of underlying neurodegenerative disorders. This is especially true in the case of hallucinatory-prone diseases like synucleinopathies. In addition, non-psychedelic drugs can bring on or exacerbate VH in PD or DLB patients. The reason for these phenomena relies on the overall impairment of multiple neurotransmitters and neural networks promoted by the degenerative processes (Carhart-Harris et al., 2012; Muthukumaraswamy et al., 2013; Carhart-Harris et al., 2016). The complexity of these neurochemical and/or network interactions are the focus of ongoing in-depth investigations aimed at identifying the distinct contribution of each neurotransmitter and neural circuitry to the production of the hallucinatory phenomena in different clinical settings.

### The Theoretical Framework: Thalamic-Driven Decoupling of the Default Mode Network

Early studies, at the beginning of the levodopa era, have proposed that the presence of VH in PD patients was the results of pharmacological treatment (Moskovitz et al., 1978; Poewe, 2003). The hypothesis was eventually disproved by studies showing no direct correlations with the levodopa administration. VH have also been linked to the presence of neuronal hyperexcitability and increased spontaneous activity occurring within the higher visual cortex; phenomena once considered to be triggered by an ongoing condition of chronic functional deafferentation (Onofrj et al., 1986; Ghilardi et al., 1988; Ffytche, 2007). The model implied the presence of dopamine-dependent visual dysfunctions but has been disproved by three lines of evidence as 1) in non-hallucinating PD subjects, visual dysfunctions are corrected by dopaminergic drugs while PD-related VH are actually triggered by the same compounds; 2) there is no increased incidence of VH in PD patients who are also affected by eye diseases; 3) the hallucinations found in PD and DLB patients can exhibit multimodal features.

**Figure 1:**
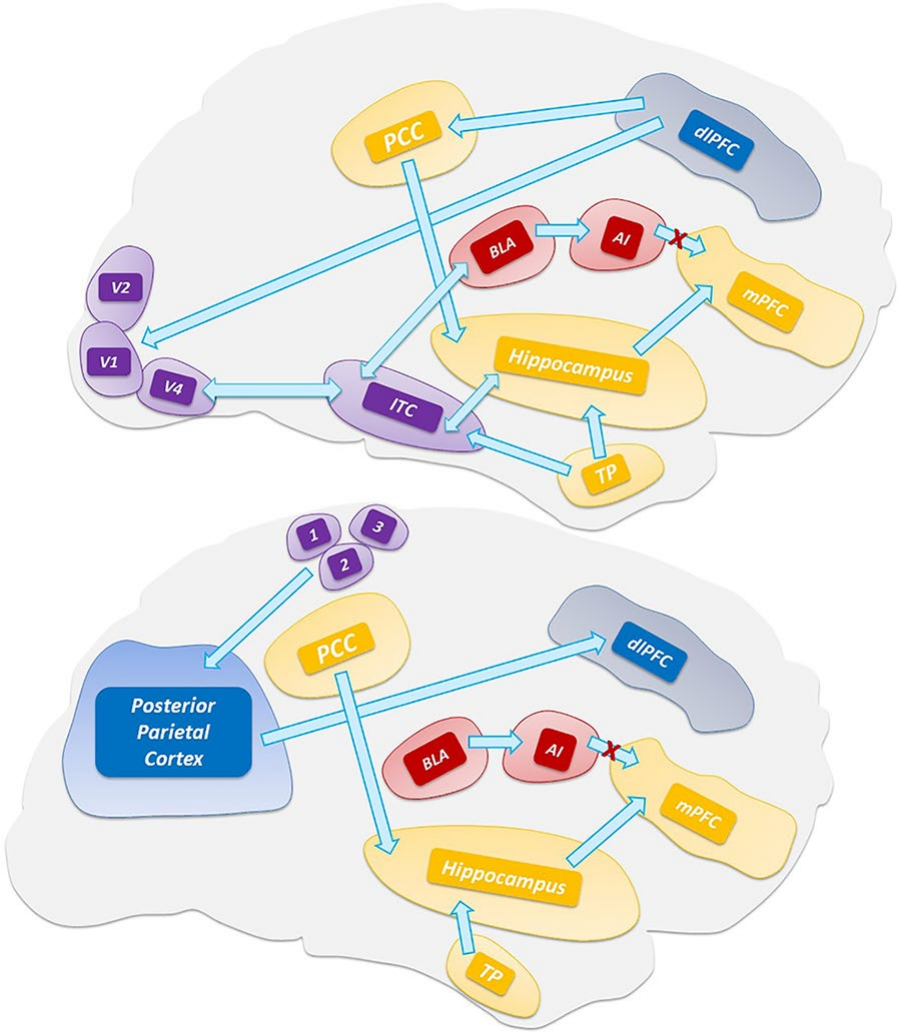
Top-down processing of perception. The scheme depicts the visual (top) and somatosensory (bottom) pathways that are involved in the activation of the default mode network and hippocampus. Abbreviations: V1, V2, V4 visual areas; ITC, inferior temporal cortex; TP, temporal pole; 3,1,2 somatosensory areas.

A more recent model for VH has considered the phenomenon as the result of a thalamic-driven derangement of the activity of top-down attentional processing of visual stimuli (**Figure 1**). The model posits that alterations of the network balance affect the brain computational processes involved in predictive coding (Friston, 2010; Sterzer et al., 2018). According to the model, VH arise when the overall prevalence of priors (i.e., the inner prior knowledge of likely candidates that match a given sensory input) over perceptions generates new percepts that are not based on matching stimuli (Clark, 2013). The process depends on the dysregulated activity of the DMN. The model further posits a sequence of events by which VH are generated when the uncontrolled activity of the DMN escapes from the modulatory activity exerted by the task-positive networks [TPNs, a set of networks like the dorsal attention network (DAN) and ventral attention network (VAN) that respond with increased activation to cognition-demanding tasks]. Thus, upon the TCD-like thalamic activity and the uncoupling from TPNs, the DMN enters in an unstable, entropic, state that unleashes the production of oneiric, dissociative, or altered state of consciousness, and ultimately VH (Muthukumaraswamy et al., 2013; Carhart-Harris et al., 2014; Carhart-Harris et al., 2016).

### The Thalamic Driver

Based on a growing body of evidences, it has also been proposed that the thalamus is the driver for these unbalanced network activities as this subcortical hub is critical to combine thalamic sensory filtering as well as the generation of thalamic rhythms associated with the thalamocortical dysrhythmia (TCD) complex (Llinàs et al., 1999 Vanneste et al., 2018). Historically, the thalamus has been viewed as a relay station for the transmission of sensory and motor information to the cortex. More recent evidence indicates that the thalamus is a key hub of the DAN, VAN, and DMN. This filtering activity, particularly in the case of somatic sensations and visual stimuli, is carried out by the first-order thalamic nuclei like the ventral posterior nucleus (VPN) and the lateral geniculate nucleus (LGN) (Ward, 2013). The thalamus exerts critical filtering within cortical-striatal-thalamo-cortical loops and the prefrontal cortex (Geyer and Vollenweider, 2008). The striatum inhibits the thalamic filtering and the dopamine- and serotonin-driven thalamic activation promotes the sensory overload to the cortex that generates cognitive disruptions, ego dissolution, and altered states of consciousness (Vollenweider, 2001a; Sherman, 2016; Halassa and Kastner, 2017). Through inputs from the associative (mediodorsal and intralaminar) and posterior (ventral-posterior and pulvinar) nuclei, the thalamus controls critical nodes of the DMN [i.e., the medial prefrontal cortex (mPFC) and posterior cingulate cortex (PCC)]. The thalamoreticular nucleus (TRN), a shell of GABA-releasing neurons surrounding the thalamic circuits (Pinault, 2004; Benarroch, 2013), receives excitatory inputs from the cortex and the dorsal thalamus and, in turn, projects inhibitory efferent inputs to the high-order thalamic nuclei (Benarroch, 2013; Ward, 2013). Hence, TRN acts as the real pacemaker of thalamocortical loops (Min, 2010). TRN oscillations are a pivotal factor for the indirect modulation of cortical activity (in particular, layers IV, V, VI) in physiological and pathological conditions (Ward, 2011). TRN also receives cholinergic inputs from the pedunculopontine nucleus (PPN) and inhibitory GABAergic inputs from the globus pallidus (Benarroch, 2013). The PPN, a brainstem nucleus consisting of cholinergic neurons, controls the thalamocortical excitability and behavioral outputs. The PPN activates the thalamocortical neurons and promotes the high-frequency cortical oscillations (burst-firing mode) associated with wakefulness.

### Default Mode Network Decoupling in the Production of Visual Hallucinations

**Figure 2:**
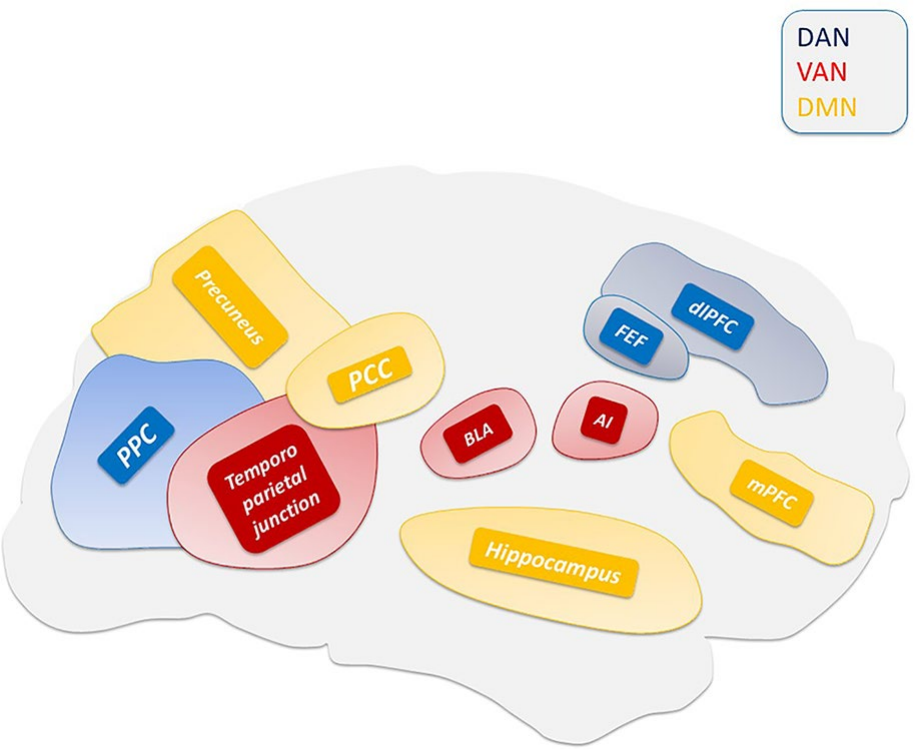
Main hubs of ventral attention system (VAN), dorsal attention system (DAN), and default mode network (DMN).

**Figure 3:**
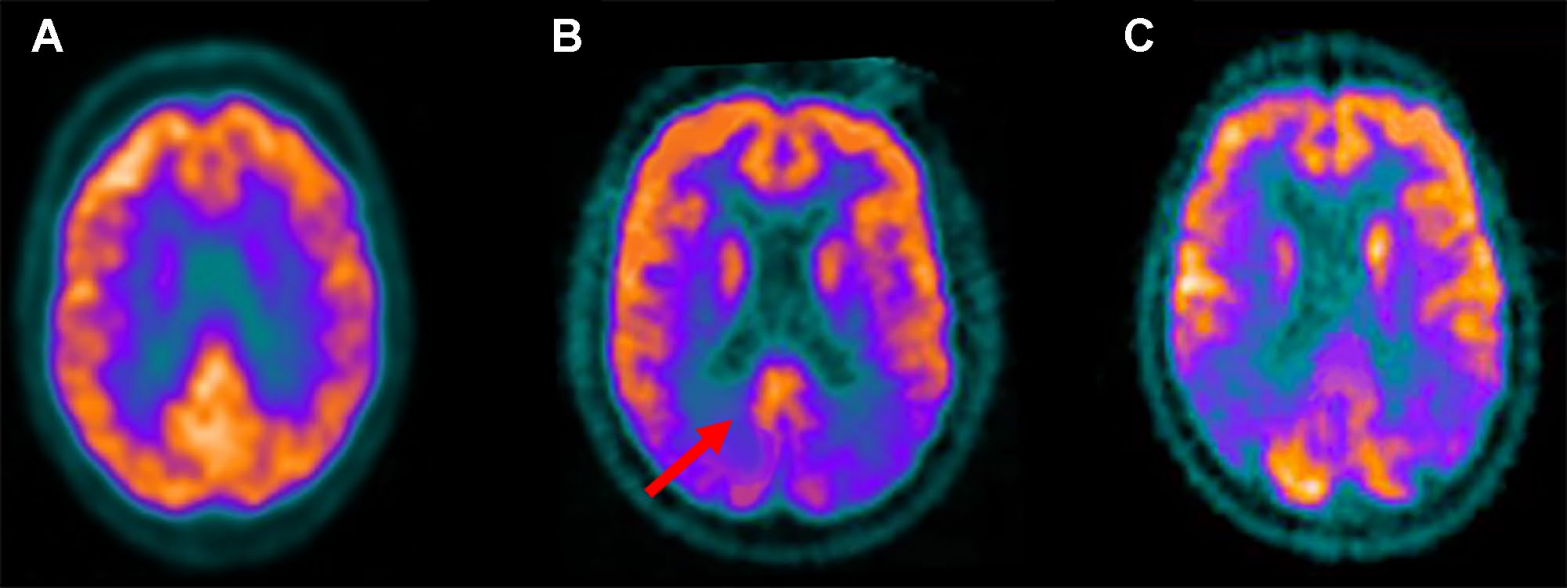
Fludeoxyglucose F 18 (18F-FDG) positron emission tomography standard axial view transacting the posterior cingulate region obtained in a healthy control **(A)**; in a patient with Lewy bodies dementia (DLB) **(B)**; in an Alzheimer disease (AD) patient **(C)**. Note that reduced 18F-FDG uptake, indicative of glucose hypometabolism and reduced synaptic activity, is present in the occipital lobes while preservation of metabolic activity is found in the posterior cingulate cortex (red arrow). This feature, known as the “cingulate island sign,” is often seen in DLB but not AD patients.

Central to the thalamic-driven model of VH is the altered functioning of the DMN. The DMN is involved in multiple functions related to self-relevant and internally directed information processing (**Figure 2**) (Andrews-Hanna et al., 2014; Raichle, 2015). The DMN is deactivated upon task execution (**Supplementary Video 1**) and exhibits high levels of activity at rest (Raichle et al., 2001). TPNs monitor and keep in check the DMN activity (Fox et al., 2005). The inverse coupling between the DMN and TPNs is at the core of the brain metastability. Alterations of the metastability lead to decreased inhibition of the DMN that promotes the intrusion of unstable perceptions (Garrity et al., 2007; Jeong and Kubicki, 2010), a process facilitated by the down-regulation of the TPNs (Wotruba et al., 2014). A critical player in the production of the network unbalance is the PCC (**Figure 3**), a region belonging to the DMN (Leech and Sharp, 2014) that exhibits transitional patterns of connectivity with TPNs. The overactivation of the dorsal PCC has been linked to the intrusion of introspective mental activities into task performances (**Supplementary Video 2**) (Weissman et al., 2006; Sonuga-Barke and Castellanos, 2007). The complexity of PCC functioning has been revealed by the experimental use of psychedelic drugs. These compounds alter the connectivity of the PCC and DMN (Muthukumaraswamy et al., 2013; Carhart-Harris et al., 2017) and generate psychedelic phenomena through the 5-hydroxytryptamine (5-HT)2A-dependent disinhibition of the thalamus and the overactivation of the PCC (Preller et al., 2019).

We and others have proposed that VH are generated by a synuclein-driven combination of thalamic- and PPN- driven dysfunctions (Henderson et al., 2000; Rub et al., 2002; Pimlott et al., 2006; Janzen et al., 2012; Onofrj et al., 2019). In the model, PPN alterations promote the TRN activation and the inhibition of the first-order nuclei of the thalamus, thereby ultimately generating pathological, “TCD-like,” theta rhythms. These theta rhythms affect the thalamic modulation of cortico-cortical loops and unleash the DMN hyperactivity. Thalamic dysfunctions synergistically act to alter the filtering of external and internal information, in a process, similar to lysergic acid diethylamide (LSD)-driven mechanisms, that inundate the primary cortex with an overload of irrelevant information (Preller et al., 2019). The altered functioning of the DMN and the visual pathways promote the incorrect inclusion of contents of visual perception into the autobiographical narrative (Shine et al., 2014; Franciotti et al., 2015; Bejr-Kasem et al., 2019). Supporting this model, PD or DLB patients suffering from VH exhibit increased DMN-driven activation of the visual cortex (Shine et al., 2015) as well as structural alterations occurring in the DAN and VAN (Delli Pizzi et al., 2014; Goldman et al., 2014; Weil et al., 2016).

## Neurotransmitters Involved in Visual Hallucinations

### The Interplay of Neurotransmitters Involved in the Physiology and Pathology of Visual Processing

The physiological modulation of visual processing requires a complex interplay among several neurotransmitters (**Table 1**). Dopaminergic, serotonergic, cholinergic, and GABAergic neurons are largely involved in these processes (Jacob and Nienborg, 2018). Second-order thalamic nuclei receive dense dopaminergic projections arising from pars compacta of the substantia nigra (SN), ventral tegmental nuclei, pontine nuclei, hypothalamus, periaqueductal gray, ventral mesencephalon, and parabrachial regions (Sánchez-González et al., 2005; Jacob and Nienborg, 2018). Several observations indicate the participation of serotonin (Marek et al., 2001; Barre et al., 2016) and noradrenaline (Morrison and Foote, 1986; Jacob and Nienborg, 2018) in the early and complex visual processing as well as in the behavioral responses (inhibition/arousal) to visual inputs. These processes are obtained by modulating the activity of the sensory cortices, TRN, and selected thalamic nuclei, as well as distinct thalamocortical circuitry (Jacob and Nienborg, 2018). Furthermore, cortico-cortical glutamatergic and GABAergic projections are the main pathways connecting the nodes of the DAN, VAN, and DMN, whereas glutamatergic projections originating from high order thalamic nuclei reach different cortical areas and are modulated by GABAergic projections sprouting from the TRN.

### Serotonergic System

Dysfunctions of serotonergic networks have been extensively described in synucleinopathies (Braak et al., 2003; Seidel et al., 2015). According to a recent and intriguing study, the appearance of pathological alterations of these networks precedes the occurrence of dopaminergic impairment, in carriers of the A53T mutation of SNCA, the gene encoding for a-synuclein (Wilson et al., 2019). The study employed a radio ligand, [¹¹C] 3-amino-4-(2-dimethylaminomethylphenylsulfanyl)-benzonitrile, to assesses with positron emission tomography (PET) the binding of serotonergic transporters along with [¹²³I] N-ω-fluoropropyl-2β-carbomethoxy-3β-(4-iodophenyl) nortropane single photon emission computed tomography (SPECT) to evaluate the functioning of dopamine transporters. It was observed that pre-symptomatic A53T SNCA carriers exhibited decreased serotonin binding but unaltered dopaminergic function. In pre-symptomatic A53T SNCA carriers, decreased serotonergic activity occurred in the raphe nuclei, caudate, putamen, thalamus, hypothalamus, and amygdala. Furthermore, in symptomatic carriers, the decreased serotoninergic binding was extended to the hippocampus, cingulate, insula, and the neocortex in conjunction with the appearance of striatal dopaminergic deficits. These data support the notion of an early dysfunction of the serotoninergic neurotransmission and they are in line with the Braak's staging hypothesis that posits a caudorostral gradient for the α-synuclein deposition. Unfortunately, the authors of the study did not mention the prevalence of VH in their cohorts.

Serotonin neurotransmission encompasses receptors that can be divided into seven subtypes (5-HT1 to 5-HT7). These receptors are all metabotropic except the ionotropic 5-HT3 receptor. 5-HT1 receptors are located mostly in raphe nuclei of the medulla oblongata, amygdala, and hippocampus (5-HT1A) where they work as somatodendritic autoreceptors modulating the monoaminergic release and neuroendocrine secretion (Pompeiano et al., 1994). 5-HT1D/1B receptors are expressed in the caudate, cortex, and SN (Hartig et al., 1992), as well as in blood vessels. 5-HT1A and 5-HT1B receptors facilitate dopamine release mainly in mesocortical and mesolimbic systems while their interaction with nigrostriatal dopaminergic projections is controversial (Alex and Pehek, 2007). 5-HT2A/C receptors are highly expressed in the prefrontal and anterior cingulate (ACC) cortices (Pazos et al., 1985; Fischette et al., 1987) as well as in the ventral tegmental area [VTA (5-HT2C)], where they modulate dopaminergic release. 5-HT2A/C receptors are strongly implicated in the production of VH (see also “*Psychedelic Drugs*”) and they are a preferential target in the treatment of PD, schizophrenia, depression, drug abuse, and anxiety. 5-HT3 and 5-HT4 receptors are expressed in the parasympathetic terminations of the gastrointestinal system where, by inducing acetylcholine release, stimulate motility. These receptor subtypes are targeted in the treatment of nausea and vomiting, despite some evidence supports the use of 5-HT3 receptor antagonists as a treatment for VH (see “*Novel Anti-Serotonergic Drugs*”) (Wilde and Markham, 1996; Morales et al., 1998). 5-HT2A cortical receptors are part of the serotonergic projections that originate from raphe nuclei. Serotonin is also involved in the antinociceptive effects, associated with the activation of descending projections arising from the periaqueductal gray.

DLB patients, showing VH, exhibit synuclein depositions in the dorsal and median raphe nuclei (Benarroch et al., 2007) as well as the reduction of serotonin levels in the striatum and frontal cortex (Perry et al., 1993a; Ohara et al., 1998; Francis, 2009). Dense serotonin innervation from dorsal raphe nuclei is also found in the intralaminar and higher-order nuclei as well as in the LGN (De Lima and Singer, 1987; Vertes et al., 1999; Varela, 2014). TRN neurons express 5-HT1A, 5-HT1B, 5-HT2A, and 5-HT2B (McCormick and Wang, 1991; Rodríguez et al., 2011; Shukla et al., 2014) receptors that affect the plasticity of thalamocortical synapses (Marek et al., 2001; Barre et al., 2016). Serotonin excites TRN neurons *via* 5-HT1C or 5-HT2 receptors (McCormick and Wang, 1991) and modulates GABA release *via* presynaptic 5-HT1A or 5-HT2A receptors (Goitia et al., 2016). Differences in the expression of receptor subtypes have been reported when comparing DLB and PD patients with or without VH. Previous studies have shown a higher 5HT2 density, as assessed with audioradiography, in DLB patients with VH (compared to patients without VH), in the cortical layers III and V of the temporal lobe (Cheng et al., 1991). Moreover, *post mortem* and *in vivo* studies performed in PD and DLB who had suffered from VH, revealed a higher density of 5-HT2A receptors within several brain regions of the ventral visual pathway (right fusiform gyrus, inferior occipital gyri), together with the inferior temporal cortex as well as in frontal regions (dorsolateral prefrontal and orbitofrontal cortices) (Huot et al., 2010). A reduction of serotonin transporter (SERT) expression within the basal ganglia (caudate nucleus > putamen) and the PCC has also been described (Politis and Niccolini, 2015). Increased serotonin signaling turnover has been demonstrated by *post mortem* studies in the brains of DLB patients (Esmaeeli et al., 2019). Finally, in the LGN, 5-HT1 presynaptic receptors negatively modulate visual inputs to thalamic neurons (Manford and Andermann, 1998), thereby inducing a deafferentation-like effect that may generate spontaneous VH.

### Dopaminergic System

The role of dopamine in the production of hallucinations has been extensively investigated in the context of schizophrenia (Miyamoto et al., 2005; Kuepper et al., 2012). The dopamine hypothesis of schizophrenia has identified a central role for the overactivation of dopaminergic neurotransmission in the mesolimbic pathway (Davis and Kahn, 1991). However, a successive revision of the theory posits that the dopaminergic activation of the nigrostriatal pathway is critical for the production of hallucinations (Kuepper et al., 2012). The dopaminergic system encompasses five (D1-5) dopamine receptor subtypes that are classified into two groups (D1- and D2-like) according to their structure and biological effects. The D1-like subtype includes the D1 and D5 receptors (D1 and D5) whereas the D2-like subtype encompasses the D2 receptors [D2; divided into two isoforms (D2-short and D2-long)], the D3 and D4 receptors (D3 and D4). All the dopamine receptors are metabotropic (Rangel-Barajas et al., 2015). The D1-like group is highly expressed in the striatum, nucleus accumbens, SN pars reticulata, and the olfactory bulb. These receptors are also moderately expressed in the entopeduncular nucleus, cerebral aqueduct, and ventricles, and they show low density in the dorsolateral prefrontal cortex, cingulate cortex, and hippocampus.

The dopamine receptors are involved in the modulation of movement as well as in cognitive functions like attention, learning, memory, and reward. D2-like receptors are mainly expressed in the striatum, external globus pallidus, nucleus accumbens, amygdala, cerebral cortex, hippocampus, and pituitary gland. These receptors are involved in movement control, impulses, and cognition (Mishra et al., 2018). Previous studies have suggested that dopaminergic projections originating from the SN pars compacta activate the D2 and D4 receptors in the TRN, thereby suppressing GABA signaling (Behrendt, 2006). Molecular investigations have indicated the presence of the D1 receptor mRNA in the intralaminar nuclei as well as in the lateral geniculate and posterior thalamic nuclei (Fremeau et al., 1991). High D2 receptor binding has been found in the midline and intralaminar nuclei, and to a lesser degree, in primary nuclei and within the TRN (Rieck et al., 2004). D2 and D4 agonists decrease the tonic inhibition induced by GABA agonists (Yagüe et al., 2013). D4 agonism diminishes the efficacy of the GABAergic inhibition exerted by the globus pallidus on the TRN (Govindaiah et al., 2010). These findings are in line with the studies proposing abnormal functioning of GABAergic/glutamatergic cortical circuits (Lewis et al., 2005) that triggers an excitatory overdrive in the modulation of the SN and increases the release of striatal dopamine thus inducing positive symptoms (Deutch, 1992; Harden et al., 1998; Kessler et al., 2009).

Thalamic abnormalities in dopaminergic neurotransmission have been reported in DLB patients. DLB patients with parkinsonism show a significant increase of D2 receptor density in the laterodorsal and ventral-intermedius nuclei, whereas DLB patients without signs of parkinsonism do not exhibit increased D2 density in any brain areas. D2 binding is significantly higher in patients with increased extrapyramidal symptoms (Piggott et al., 2007). The striatal density of the dopamine transporter (DaT) has also been found to correlate more with motor disturbances than with neuropsychiatric symptoms (Ziebell et al., 2013; Siepel et al., 2016; Shimizu et al., 2017). Changes in the striatal dopamine levels have been found to correlate with the DMN activation (Tomasi et al., 2009) and the modulation of the attention pathways (Nieoullon, 2002). High striatal DaT levels also correlate with the DMN deactivation that occurs upon the execution of attention tasks (Ojo and Fernandez, 2016). In this setting, decreased dopamine levels have been associated with lower deactivation of the parietal component of the DMN (i.e., the precuneus) and higher deactivation of the ventral ACC. A functional magnetic resonance imaging (fMRI) study analyzed the DMN activity and the effects driven by dopaminergic drugs in PD patients (Krajcovicova et al., 2012). The study demonstrated that subjects treated with a daily administration of levodopa increased the functional connectivity of the PCC and decreased the DMN activation in the left parahippocampal gyrus upon the execution of a cognitive task (Krajcovicova et al., 2012). Another study revealed that PD patients deactivate the ventral mPFC of the DMN but failed to deactivate the posterior medial and lateral areas of the network, a pattern restored by the introduction of levodopa (Delaveau et al., 2010).

### Cholinergic System

Cholinergic innervation is widespread and essential for cognition and autonomic functions. Acetylcholine works on two different receptors: muscarinic (MAchR) and nicotinic (NAchR). MAchR belong to the family of seven transmembrane receptors coupled to G-proteins (Lefkowitz, 2007). In the central nervous system (CNS), the muscarinic system plays an essential role in the regulation of many sensory, motor, and autonomic processes (Koshimizu et al., 2012) as well as in learning and memory (Coyle et al., 1983). MAchR are sub-divided into five types M1–M5 (Bonner et al., 1987). MAchR are further categorized into two groups (Hulme et al., 1990; Wess et al., 1997). The M1-like subfamily (M1, M3, and M5) is coupled to Gαq/11 protein which causes the activation of phospholipase C. Whereas the M2-like subfamily (M2 and M4) is coupled to Gi/o, which inhibits adenylate cyclase (Matsui et al., 2004). NAchR are ligand-gated ion channels formed by the assembly of five subunits, around a central pore, in homomeric or heteromeric conformation (Dani and Bertrand, 2007).

MAchR and NAchR are part of the basal forebrain and the pedunculopontine networks (Mesulam et al., 1983; Gotti and Clementi, 2004; Varela and Sherman, 2007). The basal forebrain complex arises from the basal nucleus of Meynert and projects to the hippocampus, the frontal and prefrontal regions. The pedunculopontine system projects to several thalamic and midbrain dopaminergic nuclei (Gotti et al., 2006; Carruthers et al., 2015). The cholinergic system is a critical modulator of the thalamus. The TRN expresses muscarinic (M2) and nicotinic (α7) receptors (Court et al., 1999; Carden and Bickford, 1999; Quik et al., 2000; Ni et al., 2016). Acetylcholine induces a biphasic activity in the TRN, thereby promoting an early depolarization (likely dependent on α7 activation) followed by a sustained hyperpolarization (due to M2 activation) (McCormick and Prince, 1986; Sun et al., 2013; Ni et al., 2016). Thalamocortical neurons are also influenced by the depolarizing M1 and M3 receptors and the hyperpolarizing M2 receptors (Flynn and Mash, 1993; Varela and Sherman, 2007). Intrinsic cholinergic neurons also modulate the striatum (Gotti et al., 2006). M1, M2, and M4 receptors are expressed in the striatum, along with the D2 receptors. The M5 receptor, expressed in the VTA, the hippocampus and SN, plays an essential role in favoring dopamine release, thereby facilitating dopamine-mediated behavioral responses including delirium-like sleep behavior disorders and hallucinations (Levey, 1996; Trzepacz, 1999; Trzepacz, 2000). Finally, the M3 receptor is widely involved in the regulation of the release of several neurotransmitters, including dopamine, GABA glycine, and endocannabinoids (Zhang et al., 2002; Ohno-Shosaku et al., 2003).

The degeneration of cholinergic systems and the basal forebrain network are common features of Alzheimer's disease (AD), PD, and DLB. This process affects muscarinic and nicotinic signaling (Ferreira-Vieira et al., 2016). A correlation between the neocortical loss of acetylcholinesterases (AchE) and VH has been observed in DLB (Perry et al., 1993; Perry et al., 1999; O'Brien et al., 2005). Prominent decrease of α7 expression and increased density of α4β2 NAchR in the occipital cortex have been described in these patients (Perry et al., 1990 (b); Court et al., 2001; O'Brien et al., 2008). A marked decrease (about 50%) of α7 NAchR has also been reported in the TRN, thereby offering a functional link between the cholinergic imbalance and the TCD of DLB patients (Perry et al., 1999). Despite the role of nicotinic receptors in the modulation of DLB-related psychotic symptoms, to date, drugs interacting with NAchR have not been found to impact VH. Interestingly, a recent cross-sectional study assessed the *in vivo* cholinergic activity of pre-symptomatic LRRK2 carrier PD patients (Liu et al., 2018). When compared to healthy controls, symptomatic sporadic and LRRK2 carrier PD patients, pre-symptomatic LRRK2 carriers showed a paradoxical increase of cholinergic activity occurring the thalamus and DMN-related areas, thereby challenging the notion of cholinergic network impairment in PD.

### Glutamatergic System

Glutamate is the most important excitatory neurotransmitter of the CNS. Glutamatergic synapses are involved in cortico-cortical (Roland et al., 2014), thalamo-cortical (Alexander et al., 2006), striato-cortical (Lassus et al., 2018), and hippocampal (Scherzer et al., 1998) networks that mediate key functions like motor control, pain perception, memory, and learning. Several neurological and psychiatric conditions are related to the presence of glutamatergic system dysfunction (Rothman and Olney, 1987). Glutamate binds ionotropic and metabotropic receptors. Ionotropic receptors are fast-acting and allow the influx of extracellular sodium and calcium as well as efflux of potassium ions. Ionotropic glutamate receptors are divided into three groups: α-amino-3-hydroxy-5-methyl-4-isoxazolepropionic acid (AMPA), some Kainate, and N-methyl-D-aspartate (NMDA) receptors (Traynelis et al., 2010).

AMPA receptors are widely expressed in the CNS, mainly in post-synaptic regions, where they provide sodium-mediated depolarizations. AMPA receptors are tetrameric structures made by the heteromeric assembly of four subunits (GluA1–GluA4). The GluA2 subunit controls the receptor permeability to calcium (Bochet et al., 1994; Jonas et al., 1994; Wright and Vissel, 2012). The subunit composition controls different biophysical properties and pharmacological sensitivity. Calcium-permeable AMPA receptors are present in the hippocampus, amygdala, and cerebellum where the receptors mediate plasticity processes upon brain development (Wiltgen et al., 2010) or are key players in the modulation of neurodegenerative conditions (Weiss, 2000a; Weiss, 2000b; Corona et al., 2011). The interaction between AMPA receptors and α-synuclein oligomers in pre- and post-synaptic regions have been shown to enhance the AMPA receptor-mediated excitotoxic cascade in synucleinopathies (Hüls et al., 2011).

Kainate receptors are widely distributed throughout the brain in pre- and post-synaptic regions. The receptors are assembled as a tetrameric structure containing the GluK1–3 and GluK4–5 subunits (Traynelis et al., 2010). GLUK subunits show a distinct distribution in the CNS. The GluK1-3 subunits are present in the CA3 hippocampal subregion, the striatum and the inner layers of the cortex whereas the GluK4 and GluK5 subunits show a more restricted distribution. GluK4 subunits are almost exclusively expressed in the hippocampus (Darstein et al., 2003) while GluK5 subunits are found in the striatum and the inner/outer layers of the cortex (Gallyas et al., 2003). Kainate receptors affect the NMDA receptor-independent long-term potentiation (LTP) occurring at the mossy fiber synapses of the CA3 hippocampal subregion (Lerma, 2003).

NMDA receptors possess the highest affinity for glutamate and have a tetrameric structure. Three families of NMDA receptor subunits have been so far identified: 1) NR1, 2) NR2 A-D, 3) NR3 A-B (Traynelis et al., 2010). The NR1 subunit is ubiquitously expressed in the CNS and controls neurodevelopment as well as LTP-related mechanisms (Ju and Cui, 2016). The NR2-A subunit is found in the neocortex and hippocampus, while NR2-B is primarily expressed in the forebrain (Monyer et al., 1994). In contrast, the NR2-C and NR2-D subunits are highly expressed in the cerebellum, diencephalon, and lower brain stem (Rossi et al., 2002). The NR3-A subunits are mainly expressed in the neocortex and vary upon development (Das et al., 1998; Henson et al., 2008). NR3-B are expressed in the brainstem and alpha motor neurons of the spinal cord as well as in the cerebellum and hippocampus (Fukaya et al., 2005). Although abnormal expressions of a-synuclein and NMDA receptors are frequently observed in the hippocampus of patients affected by synucleinopathies, the interaction between the two molecules remains poorly understood. However, the α-synuclein-driven internalization of hippocampal NR1 subunits is involved in the cognitive decline or functional impairment of PD and DLB patients (Chen et al., 2015).

Metabotropic glutamate receptors (mGluR 1–8 receptors) are classified in three groups depending on the binding specificity, ionic modulation, and conductance properties: group 1 (mGluR1 and mGluR5), group 2 (mGluR2 and mGluR3), and group 3 (mGluR4, mGluR6, mGluR7, and mGluR8) (Traynelis et al., 2010). Group I metabotropic receptors are largely expressed on postsynaptic membranes and have been associated with the modulation of learning, memory, addiction, and movement (Niswender and Conn, 2010). Group II metabotropic receptors are expressed in pre- and post-synaptic regions where they influence glutamate transmission. Dysfunctions in the activity of group II metabotropic receptors have been implicated in the modulation of anxiety, schizophrenia, and AD (Swanson et al., 2005). Group III metabotropic receptors are pre-synaptic and inhibit neurotransmitter release. Group III receptors are expressed in the hippocampus and hypothalamus and may play a role in PD and DLB as well as in anxiety disorders (Dalfó et al., 2004; Swanson et al., 2005). Glutamatergic synapses interact with dopaminergic and serotoninergic systems and play an important role in the genesis of auditory hallucinations (Sommer et al., 2018) and VH. In that respect, the hypofunction of NMDA receptors along with the overactivation of D2 and 5-HT receptor subtypes has been indicated to converge in producing VH and other psychotic symptoms (Rolland et al., 2014).

Moreover, according to a recent model (Grace, 2016), the NMDA receptor activation modulates the activity of parvalbumin-positive GABAergic interneurons located in the ventral subiculum. The activation of parvalbumin-pyramidal neurons is needed to drive gamma rhythmic activity, thereby providing an inhibitory input to pyramidal neurons. The NMDA receptor inhibition produces an overdrive of the nucleus accumbens which inhibits the ventral pallidum and increases the excitability of dopaminergic neurons of the lateral VTA that projects to the associative striatum, thereby generating VH. This model explains the presence of positive symptoms, including VH, in conditions like the anti-NMDA receptor autoimmune encephalitis, schizophrenia, and PD-related anxiety (Kuppuswamy et al., 2014; Barry et al., 2015).

### GABAergic System

GABA is the principal inhibitory transmitter of the CNS. GABA acts through the activation of three receptor subtypes: GABA-A (Curtis and Johnston, 1974; Johnston, 1996), GABA-B, and GABA-C (Chebib and Johnston, 1999; Tritsch et al., 2016). GABA-A and GABA-C receptors are ionotropic, whereas GABA-B is metabotropic. GABA-A receptors, which are transmembrane protein complexes made up of five subunits, produce most of the physiological actions of GABA (Sieghart et al., 1999). Many drugs modulate distinct allosteric binding sites on these receptors in order to produce clinically relevant effects in epilepsy, anxiety, and sleep disorders (Smith and Olsen, 1995). A recent study employing magnetic resonance spectroscopy revealed reduced GABA concentrations in the visual cortex of PD patients exhibiting complex VH (Firbank et al., 2018). These data are in agreement with neuropathologic findings showing reduced GABAergic activity in the visual cortex of DLB patients (Khundakar et al., 2016). DLB and PDD patients show glucose hypometabolism and hypoperfusion in the medial occipital lobe, including the primary and secondary visual cortices (Khundakar et al., 2016). The reduction in GABA levels has been suggested to serve a role in optimizing the visual processing in the presence of poor input to the visual cortex. The process can be carried out at the price of increasing the misclassification of ambiguous stimuli (Bowman et al., 2017). However, recent evidence indicates no correlations between alterations of GABA levels and the severity of VH, thereby implying that other factors, like the processing of visual stimuli and attention, play a role in favoring the generation of hallucinations (Firbank et al., 2018). Nevertheless, alterations in GABAergic transmission may contribute to the dysfunctional integration of perceptual and attentional processing, mechanisms that are involved in the production of VH (Shine, 2014).

## Pharmacological Modulators of Visual Hallucinations: Inducing Drugs

### Psychedelic Drugs

As mentioned above, the production of hallucinations and psychotic-like symptoms has been investigated by many pharmacological studies employing psychedelic drugs like LSD, mescaline, psilocybin, and ayahuasca (Geyer and Vollenweider, 2008). LSD produces visual, auditory, cenesthetic hallucinations, ego-, bodily, and time-related distortions as well as derealization and depersonalization (Nichols, 2016) (Linton, 1962). The molecule exhibits complex pharmacology and modulates serotonergic, dopaminergic, and glutamatergic transmissions. The activation of the 5-HT2A receptors in the pyramidal neurons of the prefrontal and limbic cortex is now considered the most critical mechanism for the LSD-driven production of schizophrenia-like hallucinations (Passie et al., 2008; Eggers, 2013; Kometer and Vollenweider, 2018). The LSD-driven modulation of dopaminergic neurotransmission appears to have a lesser role in the production of psychedelic effects. LSD exhibits both agonistic and antagonistic effects on D1 and D2 dopaminergic receptors although neurophysiological studies on animal models have indicated a dose-dependent LSD inhibition of the mesolimbic dopamine firing (von Hungen et al., 1974; De Gregorio et al., 2016).

Psilocybin is an alkaloid tryptamine found in mushrooms like the *Psilocybe mexicana* (Passie, 2002). *In vivo*, psilocybin is dephosphorylated to psilocin. Psilocybin induces psychedelic effects by promoting the activation of 5-HT2A, 5-HT2C, and 5-HT1A receptors (Wolbach et al., 1962). Illusions, synesthesias, altered sense of space and time, proprioceptive alterations are common effects triggered by psilocybin (Geyer and Vollenweider, 2008; Carhart-Harris et al., 2010; Turton et al., 2014). Functional neuroimaging studies, performed after psilocybin administration, have shown increased functional connectivity between the DMN and TPNs as well as decreased oscillatory activity within the PCC (Muthukumaraswamy et al., 2013; Carhart-Harris et al., 2013). The PCC exhibits a high density of 5-HT2A receptors. The decrease of the region intrinsic alpha firing rhythm correlates with the occurrence of psychedelic effects like ego disturbance and spatial and temporal distortions (Passie et al., 2008). Neuroimaging studies investigating the metabolic changes occurring after psilocybin administration have revealed the presence of thalamic hyperactivity along with the reduction in orbitofrontal and ACC metabolism in subjects showing signs of anxiety-driven ego-dissolution (Carhart-Harris et al., 2012; Lebedev et al., 2015; Nichols, 2016). The severity of visual abnormalities, including VH, has also been associated with the presence of increased metabolism in the left dorsolateral prefrontal and temporo-parietal cortex, while decreased metabolism has been reported in the parahippocampal, fusiform, and lingual gyrus (Vollenweider and Geyer, 2001b).

Ayahuasca (in the Quechua language “vine of the souls”) (McKenna et al., 1984) is a psychedelic substance employed by Amazonian native Americans to produce mystic and meditative states of consciousness (Palhano-Fontes et al., 2015). Ayahuasca is typically administered as an infusion of *Banisteriopsis caapi* liana and *Psychotria viridis* plants (Palhano-Fontes et al., 2015). While *Psychotria* contains N, N-dimethyltryptamine (DMT), *B. caapi* is enriched with psychoactive β-carbolines alkaloids like harmine, tetrahydroharmine, and harmaline (McKenna et al., 1984; Palhano-Fontes et al., 2015). DMT is an agonist of the 5-HT1A, 5-HT2A, and 5-HT2C receptors (Ray, 2010; Cameron and Olson, 2018). The compound is also endogenously synthesized from tryptophan, serotonin, or melatonin, and rapidly inactivated by monoamine oxidases (Cameron and Olson, 2018). Therefore, DMT-driven psychedelic effects can appear in subjects treated with monoamine oxidase inhibitors (MAO-I) (Palhano-Fontes et al., 2015). DMT can also bind and activate the stress-responsive sigma 1 receptors that are involved in neuroplasticity and neuromodulatory functions (Inserra, 2018). The ayahuasca effects include the production of increased introspection, VH, perceptive, affective, somatic, and cognitive changes (Riba et al., 2001). Antidepressant effects have also been reported after sporadic administration of the compound (Domínguez-Clavé et al., 2016). fMRI-based studies, performed after ayahuasca administration, have revealed decreases in DMN activity and functional connectivity occurring in the precuneus and PCC (Palhano-Fontes et al., 2015). The phenomenon has been suggested to reinforce “meditative efforts” and counteract the mind-wandering related to the DMN activation (Palhano-Fontes et al., 2015). Ayahuasca-driven increases in brain metabolism in the bilateral insular and right ACC as well as in frontal regions have been also reported (Riba et al., 2006). These findings are consistent with the enhanced emotional processing promoted by the compound (Riba et al., 2001).

### Antidepressants

Antidepressants modulate the monoaminergic neurotransmission and exhibit a prominent activity on noradrenaline and serotonin signaling. This class of compounds promotes: 1) the inhibition of the transmitter reuptake [as in the case of selective serotonin reuptake inhibitors (SSRI), serotonin-norepinephrine reuptake inhibitors (SNRI), noradrenaline reuptake inhibitors (NaRI), or tricyclic antidepressants (TCA)]; 2) the inhibition of catabolic enzymes (as in the case of MAO-I), 3) the blockade of α2-presynaptic receptors [as for noradrenergic and specific serotonergic antidepressants (NaSSA)]; 4) the enhancement of dopaminergic transmission. Atypical antidepressants combine these effects by acting synergistically on different receptor subtypes. Antidepressants exhibit unclear and contradictory effects on the production of hallucinations and psychosis.

Individual cases of patients who developed VH after receiving fluoxetine have been reported (Omar et al., 1995). Moreover, it has been suggested that the drug action on ventral striatal areas stimulates 5-HT3 receptors, promotes enhanced dopamine release from the ventral striatum, and therefore produces psychosis (Lauterbach, 1993). Antagonistic effects on 5-HT2C receptors that tonically inhibit dopamine release in nigrostriatal and mesolimbic pathways may also be involved (Alex and Pehek, 2007). TCA have consistently shown to produce hypnopompic or hypnagogic hallucinations, an effect likely due to the drug interference on the sleep architecture along with their anticholinergic effects (Cancelli et al., 2004). Amitriptyline, clomipramine, and imipramine have also found to induce VH and auditory hallucinations and worsening of psychotic symptoms (Klein, 1965; Baldessarini and Willmuth, 1968; Vallada and Gentil, 1991). Finally, it should be mentioned the possible onset of VH due to discontinuation syndrome, which can be triggered by the cessation of almost all classes of antidepressants (Yasui-Furukori N and PhD and Kanek, 2011).

### Dopaminergic Agents

Dopaminergic drugs, such as levodopa and dopamine agonists, are commonly used to manage parkinsonian motor symptoms. These agents are, however, also associated with the occurrence of significant motor and behavioral side effects. VH are sometimes a side effect of treatment (Fénelon, 2006) especially with dopamine agonists, compounds that promote an overactivation of the mesolimbic dopamine D2 and D3 receptors (Wolters, 1999; Burghauset al., 2012). While the duration and dosage of the dopaminergic therapy loosely fit with the appearance of VH (Ojo and Fernandez, 2016), VH are managed by decreasing the dosage or the use of antipsychotics.

The catechol-O-methyl-transferase (COMT) is an intracellular enzyme that catalyzes the transfer of the methyl group from S-adenosyl-L-methionine to one of the hydroxyl groups of a catechol substrate. COMT inhibitors (i.e., tolcapone, entacapone, opicapone) are widely employed to treat PD patients (Kaakkola, 2000). This class of drugs has not been directly linked to VH. However, as the molecules affect levodopa metabolism and increase its bioavailability, they may worsen the levodopa side effects, including VH (Kaakkola et al., 1994; Onofrj et al., 2001).

The Monoamine Oxidase Inhibitor B (MAOI-B) is the primary catabolizing enzyme for dopamine in the brain (Yamada and Yasuhara, 2004). Rasagiline and selegiline are two inhibitors of the MAOI-B employed in PD patients to enhance the striatal levels of dopamine. In the TVP-1012 in early monotherapy for Parkinson disease outpatients (TEMPO) study, hallucinations and confusion have been reported to occur with equal frequency in rasagiline- or placebo-treated PD patients (Siderowf et al., 2002). In the Parkinson's Rasagiline: efficacy and safety in the treatment of off (PRESTO), where rasagiline was used as an add-on to levodopa, the drug produced only a mild increase in hallucinations (4%) (Parkinson Study Group, 2005) compared to placebo (3.1%). There is conflicting evidence about the selegiline role in VH induction. Long-term selegiline-related studies have indicated that the incidence of psychotic side effects of the compound is similar to placebo (Myllylä et al., 1997). However, a sudden increase in the incidence of hallucinations has been reported in some PD patients upon the introduction of selegiline (Kamakura et al., 2004). It should be stressed that these patients, before the introduction of selegiline, were at a relatively late stage of the disease and treated with high doses of levodopa along with dopamine agonists, thereby suggesting that a complex pharmacological interaction is behind the presence of VH (Kamakura et al., 2004).

### Anticholinergic Drugs

Anticholinergic drugs are known to trigger VH (Goetz et al., 1982). The effect is well known in the case of agents with strong anti-muscarinic activity like atropine, scopolamine, and hyoscyamine. These compounds have been known for centuries to promote potent psychotropic effects, including delirium-like states, hallucinations, mood alterations, and cognitive deficits (Lakstygal et al., 2018). Atropine is extracted from *Atropa* belladonna and is a non-selective anti-muscarinic agent (Lakstygal et al., 2018). Atropine crosses the blood-brain barrier (BBB) and rapidly induces the occurrence of restlessness, memory-impairing effects, mental agitation/excitement, and hallucinations (Hammon and DeMartino, 1985; Inzelberg et al., 1990). Scopolamine is another non-selective anti-muscarinic agent that affects attention, sensory/stimulus discrimination, locomotion, and anxiety (Smythe et al., 1996; Luyten et al., 2017). The occurrence of transient psychosis has been described in patients undergoing treatment with scopolamine patches (Zhang et al., 2017). Hyoscyamine is the levorotatory form of racemic atropine and acts as a nonselective competitive antagonist of the muscarinic receptor (Soni et al., 2012). The compound generates psychomotor effects that resemble those triggered by atropine and scopolamine and include hyperactivity, motor paralysis, loss of voluntary coordination, spatiotemporal disorientation, depersonalization, memory impairment, sleep, and hallucinations/delirium-like states (Keeler and Kane, 1967). Oxybutynin, tolterodine, trospium chloride, and propriverine are other anticholinergic drugs used to treat detrusor hyperreflexia (Malkowicz et al., 1987; Gulsun et al., 2006) and associated with cognitive and behavioral side effects (Fick et al., 2003; Scheife and Takeda, 2005; Gulsun et al., 2006).

### N-Methyl-D-Aspartate Receptor Antagonists

Ketamine is a non-competitive NMDA receptor antagonist. First introduced as an anesthetic, the compound has also been employed as a recreational drug for its hallucinatory and dissociative effects (Hocking and Cousins, 2003; Correll et al., 2004; Rasmussen et al., 2013). In anesthetic and sub-anesthetic doses, the molecule produces auditory verbal hallucinations (Rasmussen, 2014). Some studies have indicated that the NMDA receptor blockade is sufficient to induce the psychomimetic effects (Pitcher et al., 2011). However, the compound also works through the activation of 5-HT2A receptors (Dall'Olio et al., 1999; Rolland et al., 2014). Ketamine affects the functional connectivity of the DMN and the sensorimotor network (Niesters et al., 2012; Driesen et al., 2013). Interestingly, ketamine shows specific, distinct, and dose-dependent effects on resting-state networks (Bonhomme et al., 2016). At lower doses, the molecule alters the connectivity of the dorsolateral prefrontal cortex, a region that is implied in the modulation of working memory while, at high doses, impairs the functional connectivity of the fronto-parietal cortex (Höflich et al., 2015).

Phencyclidine (PCP) is a non-competitive NMDA receptor antagonist that, like ketamine, induces schizophrenia-like symptoms. The two drugs are therefore also known as “dissociative anesthetics.” In addition to NMDA receptor antagonism, PCP also activates the opioid sigma-1 receptor, but the role of this interaction in the production of the hallucinogenic effects is still not completely clear (Mori and Suzuki, 2018).

Memantine is a low-affinity NMDA receptor antagonist, employed as symptomatic treatment of patients suffering from moderate to severe AD (Lorenzi et al., 2011). Memantine has been tested in PD-DLB as a neuroprotectant, but its use is significantly limited by the substantial risk of inducing psychotic side effects (Riederer et al., 1991). The majority of studies, involving DLB and PD patients treated with memantine, have indicated worsening of the psychotic symptoms, including the occurrence of delusions and hallucinations (Kim et al., 1980; Menendez-Gonzalez et al., 2005; Lipton, 2006) while only one study has shown improvement in the Neuropsychiatric Inventory scores (Ridha et al., 2005).

Amantadine, an anti-influenza drug (Liepert, 2016) and a weak NMDA receptor antagonist, is used as an add-on drug to treat the dyskinesias and freezing gait of PD patients. The pharmacodynamic properties of the drug are still unclear. The compound modulates the release of monoamines and has some neuroprotective effects (Farnebo et al., 1971; Kornhuber et al., 1994). Several reports have indicated the presence of amantadine-induced hallucinations or other psychotomimetic and neuropsychiatric side effects (Harper and Knothe, 1973; Postma and Van Tilburg, 1975; Smith, 2008; Walsh and Lang, 2012). Therefore, the use of amantadine in PD patients should be restricted to subjects with no history of psychiatric disturbances.

Propofol is mainly a GABA-A receptor agonist, but the compound also acts as an NMDA receptor antagonist. Propofol has been associated with the production of post-surgery hallucinations characterized by distinct sexual content, the occurrence of uninhibited behavior, and the verbal expression of the patient most intimate thoughts (Martínez Villar et al., 2000; Felfernig et al., 2006; Marchaisseau et al., 2008). Like ketamine, propofol affects the activity of the DMN and decreases the connectivity among the network nodes with different potency (Liu et al., 2015).

### Opioids

The opioid system is mostly involved in the regulation of pain perception (Al-Hasani and Bruchas, 2011; Stein, 2016; Valentino, 2018). Several observations have also indicated a role for opioids in the induction of VH (Sivanesan et al., 2016; Tan and Gan, 2016), although only a few studies have so far dissected the mechanisms underlying the psychoactive properties of the compounds. Early reports have associated the hallucinogenic properties of opioids with the activation of σ receptors (Sivanesan et al., 2016). However, the σ receptor is no longer considered an opioid receptor (Hayashi and Su, 2005) because it is bound by different classes of psychotropic drugs as well as by sexual hormones, haloperidol, and other substances (Hayashi and Su, 2005). The opioid receptors (OR) include the µ (MOR), κ (KOR), and δ (DOR) receptor subtypes (Al-Hasani and Bruchas, 2011; Shang and Filizola, 2015). The OR-like receptor 1 (ORL-1), also called nociceptin, has also been included in the OR family (Waldhoer et al., 2004; Al-Hasani and Bruchas, 2011). OR are coupled to G-proteins and expressed with calcium channels in the hippocampus, locus coeruleus, VTA, and dorsal root ganglia (Al-Hasani and Bruchas, 2011). The opioid-mediated regulation of synaptic transmission, obtained through a reduction of calcium currents and vesicular storage, has been described to occur after acute opioid administration (Al-Hasani and Bruchas, 2011). OR mediate the analgesic properties exerted by endogenous and most of the exogenous opioids. OR subtypes produce distinct mood alterations. MOR agonists induce euphoria (Stein, 2016), whereas DOR activation causes anxiolytic effects (Stein, 2016; Valentino and Volkow, 2018). The activation of κ receptors has been associated with the production of dysphoric feelings as well as with sedative effects (Wang et al., 2010; Maqueda et al., 2015; Valentino and Volkow, 2018).

The link between κ receptors and VH is still unclear, but increasing interest is arising in κ receptor-mediated hallucinogenic properties of salvinorin-A (Chavkin et al., 2004; Babu et al., 2008; Maqueda et al., 2015). Salvinorin-A is a κ receptor agonist that binds to the dynorphin site (Roth et al., 2002). Preclinical models have also indicated an interaction of the compound with the cannabinoid CB1 receptor (Braida et al., 2007). The molecule, a non-nitrogenous neoclerodane diterpene (Chavkin et al., 2004), has been found in the plant *Salvia divinorum* (Maqueda et al., 2015). The name of the plant originates from the Mazatecs ancient beliefs regarding the spiritual and mystic properties of *S. divinorum* (Addy, 2012). Salvinorin-A has been described to produce dose-dependent alterations in body sensation and awareness, visual-auditory, and visual-proprioceptive synesthesia, depersonalization, derealization, and mood changes as well as complex visual and auditory hallucinations (Maqueda et al., 2015). Functional studies indicate that the compound acts on the medial parietal DMN and thalamus (Maqueda et al., 2015). Morphine, fentanyl, oxycodone, and tramadol are commonly associated with the occurrence of VH (even upon drug withdrawal) (Rajabizadeh et al., 2009); however, experimental evidence is missing regarding the pathophysiological underpinnings of the phenomena (Moore, 2003; Pellegrino et al., 2013; Colak et al., 2015; Sverrisdóttir et al., 2015).

#### Other Visual Hallucinations-Inducing Drugs

Calcium blockers poorly permeate the BBB, but they have been reported to trigger hallucinations, especially in the case of verapamil and diltiazem (Kumar et al., 1988; Bushe, 1988). The underlying mechanisms are still unclear, but an inhibitory effect on mitochondrial calcium efflux and neurotransmitter release has been suggested (Matlib, 1983). Alterations in calcium signaling have been implicated in psychiatric disorders such as schizophrenia and found to affect GABAergic neurons as well as the synchronization of brain rhythms (Berridge, 2014). The activity of tyrosine hydroxylase, the rate-limiting enzyme in catecholamine synthesis, is increased by calcium chelation in dopaminergic neurons (Bushe, 1988). Delirium and hallucinations are well-known side effects triggered by the use of different groups of antibiotics. Beta-lactams, and in particular carbapenems, cause inhibition of the GABA-A receptor in the CNS as well as disinhibition of the DMN (Looijestijn et al., 2015; Veillette and Van Epps, 2016). Similar GABA-A receptor-mediated mechanisms are involved in quinolones-related hallucinations (Grill and Maganti, 2011; Chauhan et al., 2013). Trimethoprim-sulfamethoxazole may also cause VH (Stuhec, 2014) with still unclear mechanisms. Among antifungal agents, voriconazole and posaconazole have been associated with VH (Agrawal and Sherman, 2004; Imataki et al., 2008; Parkes et al., 2016). Ketoconazole has been associated with the occurrence of tactile hallucinations, and in particular delusional parasitosis, phenomena due to the antagonism of the glucocorticoid receptors and an increase in corticotropin-releasing factor levels. The latter induces a concentration-dependent increase in dopamine synthesis and dopamine dysregulation in the basal ganglia is a well-established cause of psychotic symptoms (Fisch and Lahad, 1989; Huber et al., 2007).

## Treatment Options for Visual Hallucinations

### Novel Anti-Serotonergic Drugs

5-HT receptor blockade attenuates VH and psychotic symptoms in PD and DLB patients (Meltzer et al., 2010). This mechanism is exploited by pimavanserin, an inverse agonist of 5-HT2A receptors, as well as by clozapine, quetiapine, and other atypical antipsychotic drugs. Pimavanserin negligibly binds dopaminergic, adrenergic, histaminergic, and muscarinic receptors, thereby producing an almost pure antiserotonergic effect (Cruz, 2017). These positive effects of pimavanserin in the treatment of VH occur, most likely because of the lack of D2 antagonist properties shown by the compound, without producing worsening of motor functions, sedation, or hypotensive effects (Cummings et al., 2014; Fox, 2014). Pimavanserin is preferable compared to other atypical antipsychotics for its safety profile and selective activity. Pimavanserin has been approved by the Food and Drug Administration as a first-line intervention drug for the treatment of delusions and hallucinations in PDD patients.

Ondansetron is a selective 5-HT3 receptor antagonist employed for the treatment of nausea and vomiting associated with chemotherapy, radiotherapy, anesthesia, or surgery (Wilde and Markham, 1996). Two small open-label studies investigating the use of ondansetron in PD patients found positive effects on VH and delusions (Zoldan et al., 1993; Zoldan et al., 1995). The efficacy of ondansetron against hallucinations has been associated with the blockade of the neuronal 5-HT3 receptors present in the area postrema, nucleus tractus solitarius, amygdala, and dorsal raphe nucleus (Wilde and Markham, 1996). Ondansetron does not induce worsening of motor symptoms and produces mild and reversible side effects (Zoldan et al., 1993). Ondansetron also has been successfully employed as an add-on drug to other antipsychotics for the treatment of the negative symptoms in schizophrenia (Allisoc, 2010). However, other studies have contended that these positive effects are transient and may fade away with the continuation of therapy (Eichhorn et al., 1996).

### Typical Antipsychotics

First-generation or “typical” antipsychotics, employed to manage the positive symptoms of schizophrenic patients, were designed to exert D2 receptor antagonism and inhibit the dopaminergic neurotransmission in mesocortical, mesolimbic, nigrostriatal, and tuberoinfundibular pathways. These compounds promote a postsynaptic blockade of D2 receptors in the cortex and striatum (Seeman, 2002; Stone et al., 2009). As discussed above (Perry et al., 1990, 1990a); Diederich et al., 2005), VH associated with synucleinopathies are the result of a complex monoaminergic imbalance involving dopamine and serotonin pathways (Perry et al., 1990, 1990 a), but the role of dopamine in VH is less prominent than schizophrenia. Several studies indicate that the thalamic D2 receptor density is associated more with parkinsonism than VH, a notion supported by findings indicating that the striatal DaT density correlates more with motor disturbances than neuropsychiatric symptoms (Ziebell et al., 2013; Siepel et al., 2016; Shimizu et al., 2017). The use of antipsychotics is associated with severe side effects in patients with DLB. This feature, called “severe neuroleptic sensitivity,” includes the presence of cognitive decline, parkinsonism, drowsiness, as well as the neuroleptic malignant syndrome (Ballard et al., 1998). Typical antipsychotics employed for VH may worsen the extrapyramidal symptoms of PD patients and are therefore not recommended. Moreover, compared to atypical antipsychotics like quetiapine, olanzapine, and risperidone, haloperidol has been associated with a higher mortality rate of PD patients (Weintraub et al., 2016).

### Atypical Antipsychotics

Clozapine is the prototype of the atypical antipsychotics (AA) class that encompasses olanzapine, quetiapine, and risperidone. The AA have different side effects and display different tolerability properties but share similar pharmacodynamic and pharmacokinetic characteristics. AA exhibit high-affinity inhibition of the 5-HT2 receptor, low-affinity inhibition of the D2 receptor, and 5-HT1 receptor agonism (Kapur and Seeman, 2001). The main action of AA is mediated through the interaction with 5-HT receptors (Meltzer and Massey, 2011). Because of its high tolerability in the elderly and the paucity of severe adverse effects, quetiapine is still is the most commonly prescribed antipsychotic drug in PD and PDD patients (Takahashi et al., 2003; Rabey et al., 2007; Desmarais et al., 2016). The administration of clozapine, although considered very useful (Tuunainen et al., 2002; Eng and Welty, 2010), is restricted by the presence of severe adverse effects driven by the drug anticholinergic properties (Kapur and Seeman, 2001; Tuunainen et al., 2002; Eng and Welty, 2010). Because of the lower efficacy and the increased rate of side effects, olanzapine and risperidone are less used in PD and DLB (Cummings et al., 2002; Ondo et al., 2002; Panchal and Ondo, 2018).

### Acetylcholinesterase Inhibitors

Acetylcholinesterase inhibitors (AchE-I), such as donepezil, rivastigmine, and galantamine, inhibit the breakdown of acetylcholine, thereby potentiating cholinergic neurotransmission (Colović et al., 2013). AchE-I are employed in mild to moderate AD and produce transient ameliorating effects in cognition and behavioral symptoms, including VH (Onofrj et al., 2003; Birks, 2006; Ferreira-Vieira, 2016). AchE-I have also been employed in PD patients showing cognitive impairment and/or apathy or in DLB patients with VH (McKeith, 2000; Burn et al., 2006; Poewe et al., 2006). PDD and DLB patients exhibit relative hypoperfusion in the parietal and occipital brain regions (Collerton et al., 2005) and the AchE-I-driven increased perfusion of the PCC has been suggested to play a role in improving the attention to visual stimuli and decrease the VH frequency in these subjects (McKeith et al., 2004; O'Brien et al., 2005). Finally, AA that are effective in treating DLB hallucinations and also promote an increase of extracellular acetylcholine levels (Shirazi-Southall et al., 2002).

#### Other Possible Treatments for Visual Hallucinations

The use of antidepressants has been often associated with VH induction. However, a study carried out on a cohort of 692 patients affected by either AD or vascular and/or mixed dementia found that citalopram and sertraline show some efficacy against agitation or psychosis (Seitz et al., 2011). Within the atypical antidepressants, mirtazapine, a compound most likely acting *via* antagonism of the α2, 5-HT2, and 5-HT3 receptors, has been successfully employed to treat VH and auditory hallucinations in PD patients (Golden et al, 1998; Nagata et al., 2013; Tagai et al., 2013).

Vortioxetine, one of the newest antidepressants, is a SRI. The compound is also an antagonist of 5-HT1D, 5-HT3, and 5-HT7 receptors as well as agonist of 5-HT1A and partial agonist of 5-HT1B receptors (D'Agostino et al., 2015; Stahl, 2015). Vortioxetine also exhibit cholinergic activity that may be indicated for synucleinopathy-associated patients showing VH and affective disturbances (Smart et al., 2018).

No worsening of VH has been reported in advanced PD patients treated with apomorphine (García Ruiz et al., 2008; Borgemeester et al., 2016), a drug with antipsychotic effects (Smith, 1977; Tamminga et al., 1978). Apomorphine is a potent dopamine agonist with high affinity for the D1- and D2-like receptors (Millan et al., 2002). The antipsychotic activity is likely due to the presence of a piperidine moiety, which has antagonist activity on serotonergic receptors and the 5-HT2A-receptor subtype in particular (Ellis et al., 1997).

Finally, naloxone, an opioid antagonist, is effective in abrogating auditory and VH in schizophrenic patients (Gunne et al., 1977), findings that are consistent with the role of the endogenous opioid system in the induction and resolution of VH.

## Conclusions

VH are common features of PD and DLB (Halliday et al., 2011). Many questions on the pathophysiology of these phenomena are still open. Early observations suggesting an iatrogenic origin for the VH found in synucleinopathies (Moskovitz et al., 1978) have been now disconfirmed by the fact that in many cases VH appear with appropriate therapeutic courses or even in the absence of any pharmacological therapy. Moreover, several findings have also confirmed the notion that, in PD and DLB patients, the synuclein-driven deregulation of brain circuits and functional networks predispose to VH.

The development of concepts as “metastability” of cognition and “entropic brain,” proposed by Carhart-Harris and Coll. (Carhart-Harris et al., 2014), has been crucial milestone of the research in the field. This notion has indeed allowed a better understanding of the link between the TCD, already observed by Llinàs and Coll. in the 90s (Llinàs et al., 1999) and the development of the clinical symptoms exhibited by PD or DLB patients, an array ranging from cognitive fluctuations to VH. These findings have led to posit the current model of VH in synucleinopathies (Onofrj et al., 2019) which are considered to result from PPN-driven alterations of thalamic pacing due to the enhanced activity of TRN. The derangement of thalamocortical loops provokes the unleashing of the DMN that becomes disentangled from the control of the TPNs.

Several neurotransmitters have been implied in the development of VH. Serotonergic agents, like psychedelic drugs, have been proven to actively participate in the phenomenon also in healthy subjects (Geyer and Vollenweider, 2008). In particular, the activation of 5HT2A receptors which are expressed with high density in the PCC, a hub of DMN, has been strongly linked with the VH onset (Passie et al., 2008; Carhart-Harris et al., 2010). Alterations of the serotonergic system are a typical finding in synucleinopathies (Seidel et al., 2015; Wilson et al., 2019). Moreover, antipsychotic drugs modulating serotonergic transmission are now widely employed to treat VH, especially in PD and DLB patients (Carrarini et al., 2019). Further investigations will be nevertheless needed to clarify the fine downstream mechanism involved in these processes.

In addition, cholinergic impairment is also exerting a major role (Ferreira-Vieira et al., 2016) as muscarinic and nicotinic receptors have been found to be expressed in the thalamus, and in particular within the TRN (Quik et al., 2000; Ni et al., 2016). Cholinergic transmission may influence the onset and maintenance of TCD, thereby favoring VH (Varela and Sherman, 2007). In that context, AchE-I by increasing the extracellular levels of acetylcholine may be of therapeutic value and reduce VH in DLB patients (Shirazi-Southall et al., 2002), an effect also possibly due to ameliorating PCC perfusion (McKeith et al., 2004; O'Brien et al., 2005). On the other hand, anticholinergic drugs, as atropine can trigger VH (Goetz et al., 1982) and should, therefore, avoided in elderly patients when there is a suspect of underlying synucleinopathies.

A special role is played by the alterations of the dopaminergic system, the main target of the disruptive effect of a-synuclein. Although dopamine replacement therapy is a pillar for the treatment of the motor symptoms of PD and DLB, the neurotransmitter unfortunately also exacerbates some neuropsychiatric disorders associated with the disease like VH, impulse control disorders, and overall behavioral changes (Fénelon, 2006). It is now clear that dopamine therapy, especially at high dosage, can trigger VH by acting on the nigrostriatal pathway (Kuepper et al., 2012). Careful management of dopamine replacement therapy is therefore required to prevent VH onset and often significant results are obtained by decreasing the drug dosage. If that maneuver does not reach the goal, the use of AA may help (Eng and Welty, 2010; Desmarais et al., 2016).

The modulation of glutamatergic or opioid neurotransmitters is also involved in VH (Niesters et al., 2012; Sivanesan et al., 2016) but the exact activity of these systems is still largely unexplored. In summary, the neuropharmacology of VH is a fascinating and still “under construction” chapter of clinical neuroscience. The field is in need of additional functional and structural studies to shed light on a phenomenon that represents a clinical challenge and often an unmet therapeutic need.

## Author Contributions

All authors contributed to manuscript preparation, editing, and revision.

## Conflict of Interest

MO has served on the scientific advisory boards of GlaxoSmithKline, Novartis, Lundbeck, Eisai, Valeant, Medtronic, and Newron; has received speaker honoraria from Zambon, the World Parkinson Congress, the Movement Disorder Society, and the Atypical Dementias congress; publishing royalties from Springer; was an invited guest and lecturer for the Mental Disorders in Parkinson Disease Congress; serves on the editorial board of Medicine (Baltimore) and Frontiers in Neurology; has been employed as a speaker for Boehringer Ingelheim, GlaxoSmithKline, UCB, and Zambon; and has received research support from the Italian Ministry of Health and the Italian Ministry of Education. SS serves as associate editor of Frontiers in Neuroscience, Frontiers in Psychiatry, PlosOne, and Scientific Reports and is supported by non-profit agencies [the Italian Ministry of Health, the AIRAlzh Onlus (ANCC-COOP), the Alzheimer's Association – Part the Cloud: Translational Research Funding for Alzheimer's Disease (18PTC-19-602325) and the Alzheimer's Association – GAAIN Exploration to Evaluate Novel Alzheimer's Queries (GEENA-Q-19-596282)].

The remaining authors declare that the research was conducted in the absence of any commercial or financial relationships that could be construed as a potential conflict of interest.

## List Of Abbreviations

5-HT: 5-hydroxytryptamine
[¹¹C] DASB [¹¹C]: 3-amino-4-(2-dimethylaminomethylphenylsulfanyl) -benzonitrile,
[¹²³I] FP-CIT [¹²³I]: N-ω-fluoropropyl- 2β-carbomethoxy- 3β-(4-iodophenyl) nortropane
AA: atypical antipsychotic
ACC: anterior cingulate cortex
AD: Alzheimer’s disease
AchE: acetylcholinesterase
AchE-I: acetylcholinesterase inhibitors
AD: Alzheimer’s Disease
AMPA: α-amino-3-hydroxy-5-methyl-4-isoxazolepropionic acid
BBB: blood-brain barrier
CNS: central nervous system
COMT: Catechol-O-methyl-transferase
DAN: Dorsal Attention Network
DaT: dopamine transporter
DLB: Dementia with Lewy Bodies
DMT N,: N-Dimethyltryptamine
DMN: Default Mode Network
DOR: δ opioid receptor
fMRI: functional Magnetic Resonance Imaging
GABA: gamma aminobutyric acid
KOR: κ opioid receptor
LGN: lateral geniculate nucleus
LSD: lysergic acid diethylamide
LTP: long-term potentiation
MAchR: muscarinic receptor for acetylcholine
MAO-I: Monoamine Oxidase Inhibitors
MAOI-B: Inhibitor of monoamine oxidase B
MOR: μ opioid receptor
mPFC: medial prefrontal cortex
NAchR: nicotinic receptor for acetylcholine
NaRI: Noradrenaline Reuptake Inhibitors
NaSSA: Noradrenergic and Specific Serotonergic Antidepressants
NMDA: N-methyl-D-aspartate
OR: opioid receptors
PCC: posterior cingulate cortex
PCP: Phencyclidine
PD: Parkinson’s disease
PDD: Parkinson’s Disease with Dementia
PET: Positron Emission Tomography
PPN: pedunculopontine nucleus
SERT: serotonin transporter
SN: Substantia Nigra
SNRI: Serotonin-Norepinephrine Reuptake Inhibitors
SPECT: Single Photon Emission Computed Tomography
SRI: Serotonin Reuptake Inhibitor
SSRI: Selective Serotonin Reuptake Inhibitors
TCA: Tricyclic antidepressant
TCD: ThalamoCortical Dysrhythmia
TPNs: Task-Positive Networks
TRN: thalamoreticular nucleus
VAN: Ventral Attention Network
VH: Visual Hallucinations
VPN: Ventral posterior nucleus
VTA: ventral tegmental area

## Supplementary Material

Video 1 Metastability in cognition: lower entropy state. Coupled-stable, reality checking networks.

Video 2 Metastability in cognition: higher entropy state. Decoupled unstable PCC, randomly connecting motifs.
